# Bis[4-(4-pyridyl­meth­oxy)phenol-κ*N*]silver nitrate monohydrate

**DOI:** 10.1107/S1600536810020945

**Published:** 2010-06-05

**Authors:** Hong-Sen Zhang

**Affiliations:** a Modern Analysis, Test and Research Center, Heilongjiang Institute of Science and Technology, Harbin 150027, People’s Republic of China

## Abstract

In the title compound, [Ag(C_12_H_11_NO_2_)_2_]NO_3_·H_2_O, the Ag^I^ ion is coordinated by two N atoms from two different 4-(4-pyridyl­meth­oxy)phenol ligands, generating a nearly linear coordination geometry with an N—Ag—N angle of 167.1 (1)°. A three-dimensional supra­molecular network is built from the uncoordinated nitrate anion, the water mol­ecule and the cation through O—H⋯O hydrogen bonds.

## Related literature

For the synthesis of the title ligand, see: Gao *et al.* (2006 ▶); Zou *et al.* (2009 ▶). For backround to metal-organic complexes with flexible pyridyl-based ligands, see: Fun *et al.* (1999 ▶); Liu *et al.* (2010 ▶); You *et al.* (2009 ▶).
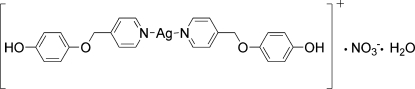


## Experimental

### 

#### Crystal data

[Ag(C_12_H_11_NO_2_)_2_]NO_3_·H_2_O
*M* *_r_* = 590.33
Monoclinic, 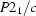

*a* = 9.458 (4) Å
*b* = 13.507 (7) Å
*c* = 20.274 (7) Å
β = 111.986 (18)°
*V* = 2401.6 (18) Å^3^
*Z* = 4
Mo *K*α radiation
μ = 0.89 mm^−1^
*T* = 291 K
0.21 × 0.19 × 0.18 mm

#### Data collection

Rigaku R-AXIS RAPID diffractometer
Absorption correction: multi-scan (*ABSCOR*; Higashi, 1995 ▶) *T*_min_ = 0.832, *T*_max_ = 0.857
22423 measured reflections
5442 independent reflections
2900 reflections with *I* > 2σ(*I*)
*R*_int_ = 0.058

#### Refinement

*R*[*F* ^2^ > 2σ(*F* ^2^)] = 0.054
*wR*(*F* ^2^) = 0.115
*S* = 1.04
5442 reflections
325 parameters
H-atom parameters constrained
Δρ_max_ = 0.28 e Å^−3^
Δρ_min_ = −0.38 e Å^−3^

### 

Data collection: *RAPID-AUTO* (Rigaku, 1998 ▶); cell refinement: *RAPID-AUTO*; data reduction: *CrystalClear* (Rigaku/MSC, 2002 ▶); program(s) used to solve structure: *SHELXS97* (Sheldrick, 2008 ▶); program(s) used to refine structure: *SHELXL97* (Sheldrick, 2008 ▶); molecular graphics: *SHELXTL* (Sheldrick, 2008 ▶); software used to prepare material for publication: *SHELXL97*.

## Supplementary Material

Crystal structure: contains datablocks I, global. DOI: 10.1107/S1600536810020945/ng2780sup1.cif

Structure factors: contains datablocks I. DOI: 10.1107/S1600536810020945/ng2780Isup2.hkl

Additional supplementary materials:  crystallographic information; 3D view; checkCIF report

## Figures and Tables

**Table 1:** Hydrogen-bond geometry (Å, °)

*D*—H⋯*A*	*D*—H	H⋯*A*	*D*⋯*A*	*D*—H⋯*A*
O1—H1*A*⋯O8^i^	0.82	1.90	2.661 (4)	155
O3—H3⋯O7	0.82	1.88	2.698 (5)	176
O8—H31⋯O7	0.85	2.05	2.885 (5)	167
O8—H32⋯O3^ii^	0.85	2.00	2.833 (4)	165

Symmetry codes: (i) 
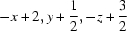
; (ii) 
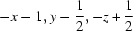
.

**Fig. 1.**
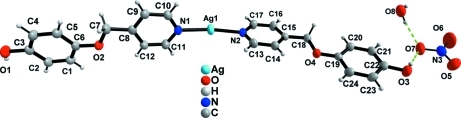
The molecular structure of title compound, showing the atom-labelling scheme and displacement ellipsoids drawn at the 30% probability level.

**Fig. 2.**
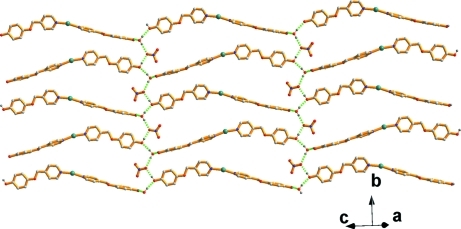
The crystal packing diagram of title compound, viewed along a axis. Dashed lines indicate hydrogen bonds, noinvolving hrdrogen atoms are omited for clarity.

## supplementary crystallographic information

### Comment

The metal-organic compounds constructed by the pyridine-containing ligands have atracted more attention for their novel and virous structures and potential applications (Fun *et al.* 1999; Liu *et al.* 2010]. A polynuclear silver(I) complex with 2-hydroxypyridine was synthesized, and the complex maybe served as an efficient urease inhibitor (You *et al.* 2009). Based on above researches, the title compound was synthesized by reacting pyridine-containing ligand with the AgNO_3_.

X-ray single-crystal analysis of title compound shows that the Ag^I^ is coordinated by two N atoms from two different 4-(4-pyridylmethoxy)-phenol ligands to generate a linear coordination geometry with the N—Ag—N angle of 167.06 (14) ° (Figure 1, Table 1). In each asymmetrical unit, the planes of the pyridine rings and benzene rings are nearly parallel and make dihedral angles of 8.462 (4)° and 7.165 (21) °. But the two ligands are vertical with the dihedral angle of two pyridine rings being 86.779 (11) °.

Two terminal hydroxyl groups, one uncoordinate water and one nitrate ion are linked together to form a three dimensional network through intermolecular O—H···O hydrogen bonds (Figure 2, Table 2).

### Experimental

The synthesis of ligand see the literature (Gao *et al.* 2006; Zou *et al.* 2009). A solution of AgNO_3_ (0.017 g, 0.10 mmol) in water (2 ml) was dropped slowly into a methanol solution (5 ml) of ligand (0.040 g, 2 mmol) to give a clear solution. Colourless block crystals of title were obtained by slow evaporation of the clear solution under room temperature after a week.

### Refinement

H atoms bound to C atoms and hydroxyl groups were placed in calculated positions and treated as riding on their parent atoms, with C—H = 0.93 Å(aromatic C), C—H = 0.97 Å (methene C), and with *U*_iso_(H) = *1.2U*_eq_(C). The H atoms of water moleule were initially located in a difference Fourier map, but they were treated as riding on their parent atoms with O—H = 0.85 Å and with *U*_iso_(H) = 1.5 *U*_eq_(O).

### Crystal data

**Table:**

[Ag(C_12_H_11_NO_2_)_2_]NO_3_·H_2_O	*F*(000) = 1200
*M**_r_* = 590.33	*D*_x_ = 1.633 Mg m^−^^3^
Monoclinic, *P*2_1_/*c*	Mo *K*α radiation, λ = 0.71073 Å
Hall symbol: -P 2ybc	Cell parameters from 11930 reflections
*a* = 9.458 (4) Å	θ = 3.0–27.5°
*b* = 13.507 (7) Å	µ = 0.89 mm^−^^1^
*c* = 20.274 (7) Å	*T* = 291 K
β = 111.986 (18)°	Block, colorless
*V* = 2401.6 (18) Å^3^	0.21 × 0.19 × 0.18 mm
*Z* = 4	

### Data collection

**Table:**

Rigaku R-AXIS RAPID diffractometer	5442 independent reflections
Radiation source: fine-focus sealed tube	2900 reflections with *I* > 2σ(*I*)
graphite	*R*_int_ = 0.058
ω scan	θ_max_ = 27.5°, θ_min_ = 3.0°
Absorption correction: multi-scan (*ABSCOR*; Higashi, 1995)	*h* = −12→12
*T*_min_ = 0.832, *T*_max_ = 0.857	*k* = −17→17
22423 measured reflections	*l* = −23→26

### Refinement

**Table:**

Refinement on *F*^2^	Primary atom site location: structure-invariant direct methods
Least-squares matrix: full	Secondary atom site location: difference Fourier map
*R*[*F*^2^ > 2σ(*F*^2^)] = 0.054	Hydrogen site location: inferred from neighbouring sites
*wR*(*F*^2^) = 0.115	H-atom parameters constrained
*S* = 1.04	*w* = 1/[σ^2^(*F*_o_^2^) + (0.0406*P*)^2^ + 0.9527*P*] where *P* = (*F*_o_^2^ + 2*F*_c_^2^)/3
5442 reflections	(Δ/σ)_max_ = 0.001
325 parameters	Δρ_max_ = 0.28 e Å^−^^3^
0 restraints	Δρ_min_ = −0.38 e Å^−^^3^

### Special details

**Table:**

Geometry. All e.s.d.'s (except the e.s.d. in the dihedral angle between two l.s. planes) are estimated using the full covariance matrix. The cell e.s.d.'s are taken into account individually in the estimation of e.s.d.'s in distances, angles and torsion angles; correlations between e.s.d.'s in cell parameters are only used when they are defined by crystal symmetry. An approximate (isotropic) treatment of cell e.s.d.'s is used for estimating e.s.d.'s involving l.s. planes.
Refinement. Refinement of *F*^2^ against ALL reflections. The weighted *R*-factor *wR* and goodness of fit *S* are based on *F*^2^, conventional *R*-factors *R* are based on *F*, with *F* set to zero for negative *F*^2^. The threshold expression of *F*^2^ > σ(*F*^2^) is used only for calculating *R*-factors(gt) *etc*. and is not relevant to the choice of reflections for refinement. *R*-factors based on *F*^2^ are statistically about twice as large as those based on *F*, and *R*- factors based on ALL data will be even larger.

### Fractional atomic coordinates and isotropic or equivalent isotropic displacement parameters (Å^2^)

**Table:**

	*x*	*y*	*z*	*U*_iso_*/*U*_eq_	
Ag1	0.90730 (4)	0.49819 (3)	0.766410 (16)	0.07746 (16)	
O1	2.1641 (3)	0.7496 (2)	1.22381 (14)	0.0838 (9)	
H1A	2.2314	0.7453	1.2076	0.126*	
O2	1.6021 (3)	0.6618 (2)	1.01902 (13)	0.0673 (7)	
O3	−0.3575 (3)	0.6172 (2)	0.27642 (14)	0.0818 (9)	
H3	−0.3967	0.5657	0.2566	0.123*	
O4	0.2108 (3)	0.5574 (2)	0.48365 (13)	0.0648 (7)	
N1	1.0938 (4)	0.5514 (3)	0.85577 (17)	0.0599 (8)	
N2	0.7030 (4)	0.4783 (3)	0.67566 (16)	0.0605 (9)	
C1	1.8626 (4)	0.6955 (3)	1.0520 (2)	0.0616 (10)	
H1	1.8487	0.6892	1.0043	0.074*	
C2	2.0045 (4)	0.7167 (3)	1.1011 (2)	0.0622 (10)	
H2	2.0861	0.7248	1.0866	0.075*	
C3	2.0268 (4)	0.7261 (3)	1.1721 (2)	0.0596 (10)	
C4	1.9048 (5)	0.7140 (3)	1.1922 (2)	0.0662 (11)	
H4	1.9188	0.7203	1.2399	0.079*	
C5	1.7617 (4)	0.6927 (3)	1.14314 (19)	0.0625 (11)	
H5	1.6802	0.6847	1.1578	0.075*	
C6	1.7397 (4)	0.6832 (3)	1.07254 (19)	0.0557 (10)	
C7	1.4773 (4)	0.6405 (3)	1.03879 (19)	0.0600 (10)	
H7A	1.4508	0.6985	1.0600	0.072*	
H7B	1.5037	0.5873	1.0735	0.072*	
C8	1.3450 (4)	0.6102 (3)	0.97345 (19)	0.0528 (9)	
C9	1.2098 (4)	0.5805 (3)	0.9801 (2)	0.0617 (11)	
H9	1.2015	0.5800	1.0244	0.074*	
C10	1.0896 (4)	0.5522 (3)	0.9206 (2)	0.0625 (10)	
H10	1.0001	0.5323	0.9257	0.075*	
C11	1.2229 (5)	0.5806 (3)	0.8502 (2)	0.0723 (12)	
H11	1.2284	0.5804	0.8053	0.087*	
C12	1.3483 (4)	0.6109 (3)	0.9068 (2)	0.0631 (11)	
H12	1.4354	0.6318	0.8998	0.076*	
C13	0.6144 (5)	0.5569 (4)	0.6534 (2)	0.0676 (11)	
H13	0.6493	0.6168	0.6763	0.081*	
C14	0.4742 (4)	0.5559 (3)	0.5987 (2)	0.0620 (10)	
H14	0.4168	0.6136	0.5854	0.074*	
C15	0.4202 (4)	0.4684 (3)	0.56382 (18)	0.0508 (9)	
C16	0.5105 (5)	0.3864 (3)	0.5868 (2)	0.0653 (11)	
H16	0.4781	0.3255	0.5650	0.078*	
C17	0.6492 (5)	0.3944 (4)	0.6421 (2)	0.0677 (11)	
H17	0.7085	0.3376	0.6568	0.081*	
C18	0.2680 (4)	0.4611 (3)	0.50477 (19)	0.0562 (10)	
H18A	0.2775	0.4262	0.4648	0.067*	
H18B	0.1983	0.4245	0.5206	0.067*	
C19	0.0684 (4)	0.5651 (3)	0.43111 (18)	0.0536 (9)	
C20	−0.0174 (4)	0.4872 (3)	0.3943 (2)	0.0585 (10)	
H20	0.0204	0.4230	0.4042	0.070*	
C21	−0.1600 (4)	0.5033 (3)	0.34252 (19)	0.0604 (10)	
H21	−0.2179	0.4500	0.3178	0.072*	
C22	−0.2163 (4)	0.5970 (4)	0.32752 (19)	0.0611 (11)	
C23	−0.1309 (5)	0.6762 (3)	0.3639 (2)	0.0657 (11)	
H23	−0.1688	0.7403	0.3537	0.079*	
C24	0.0111 (5)	0.6598 (3)	0.4155 (2)	0.0644 (11)	
H24	0.0690	0.7132	0.4402	0.077*	
O5	−0.6790 (5)	0.5495 (3)	0.15883 (18)	0.1028 (11)	
O6	−0.7139 (5)	0.3980 (4)	0.1501 (3)	0.169 (2)	
O7	−0.4972 (4)	0.4493 (3)	0.21269 (17)	0.0924 (10)	
N3	−0.6322 (5)	0.4659 (4)	0.1726 (2)	0.0748 (11)	
O8	−0.4291 (3)	0.2671 (2)	0.29281 (18)	0.0947 (10)	
H31	−0.4422	0.3165	0.2651	0.142*	
H32	−0.4804	0.2184	0.2694	0.142*	

### Atomic displacement parameters (Å^2^)

**Table:**

	*U*^11^	*U*^22^	*U*^33^	*U*^12^	*U*^13^	*U*^23^
Ag1	0.0540 (2)	0.1015 (3)	0.0613 (2)	−0.01044 (19)	0.00368 (15)	−0.00670 (19)
O1	0.0576 (18)	0.114 (3)	0.0640 (17)	−0.0152 (17)	0.0045 (15)	−0.0085 (16)
O2	0.0477 (16)	0.094 (2)	0.0534 (15)	−0.0097 (15)	0.0109 (13)	0.0040 (14)
O3	0.0646 (18)	0.097 (2)	0.0640 (17)	0.0063 (17)	0.0008 (15)	0.0130 (16)
O4	0.0518 (17)	0.0639 (19)	0.0626 (16)	−0.0080 (14)	0.0031 (14)	0.0036 (14)
N1	0.049 (2)	0.061 (2)	0.059 (2)	−0.0014 (17)	0.0082 (16)	−0.0005 (16)
N2	0.050 (2)	0.078 (3)	0.0505 (18)	−0.0069 (18)	0.0148 (16)	−0.0006 (17)
C1	0.060 (3)	0.071 (3)	0.054 (2)	−0.004 (2)	0.021 (2)	0.0004 (19)
C2	0.050 (2)	0.070 (3)	0.062 (2)	−0.003 (2)	0.016 (2)	0.000 (2)
C3	0.048 (2)	0.056 (3)	0.061 (2)	−0.0021 (19)	0.005 (2)	0.0006 (19)
C4	0.063 (3)	0.080 (3)	0.049 (2)	−0.004 (2)	0.015 (2)	−0.004 (2)
C5	0.055 (2)	0.076 (3)	0.054 (2)	−0.002 (2)	0.017 (2)	0.001 (2)
C6	0.048 (2)	0.055 (3)	0.054 (2)	−0.0013 (18)	0.0072 (19)	0.0030 (18)
C7	0.046 (2)	0.071 (3)	0.057 (2)	−0.004 (2)	0.0127 (19)	−0.0061 (19)
C8	0.051 (2)	0.044 (2)	0.057 (2)	0.0041 (18)	0.0119 (18)	−0.0015 (17)
C9	0.054 (2)	0.074 (3)	0.054 (2)	−0.003 (2)	0.017 (2)	0.001 (2)
C10	0.049 (2)	0.072 (3)	0.061 (2)	−0.004 (2)	0.014 (2)	0.002 (2)
C11	0.066 (3)	0.096 (4)	0.052 (2)	−0.008 (3)	0.018 (2)	−0.002 (2)
C12	0.055 (2)	0.075 (3)	0.057 (2)	−0.012 (2)	0.017 (2)	−0.003 (2)
C13	0.066 (3)	0.067 (3)	0.062 (2)	−0.015 (2)	0.015 (2)	−0.009 (2)
C14	0.054 (3)	0.061 (3)	0.063 (2)	−0.005 (2)	0.013 (2)	0.001 (2)
C15	0.046 (2)	0.063 (3)	0.0435 (19)	−0.0065 (18)	0.0169 (17)	−0.0004 (17)
C16	0.060 (3)	0.063 (3)	0.061 (2)	0.002 (2)	0.010 (2)	−0.010 (2)
C17	0.062 (3)	0.074 (3)	0.057 (2)	0.005 (2)	0.012 (2)	−0.001 (2)
C18	0.052 (2)	0.065 (3)	0.049 (2)	−0.005 (2)	0.0150 (19)	−0.0022 (18)
C19	0.046 (2)	0.065 (3)	0.048 (2)	−0.009 (2)	0.0151 (18)	0.0033 (19)
C20	0.055 (2)	0.058 (3)	0.057 (2)	0.001 (2)	0.0141 (19)	−0.0002 (19)
C21	0.059 (2)	0.071 (3)	0.0472 (19)	−0.010 (2)	0.0144 (18)	−0.006 (2)
C22	0.056 (3)	0.079 (3)	0.044 (2)	0.000 (2)	0.0134 (19)	0.008 (2)
C23	0.072 (3)	0.057 (3)	0.063 (2)	0.006 (2)	0.019 (2)	0.011 (2)
C24	0.065 (3)	0.061 (3)	0.057 (2)	−0.009 (2)	0.010 (2)	0.002 (2)
O5	0.110 (3)	0.107 (3)	0.076 (2)	0.020 (3)	0.017 (2)	0.013 (2)
O6	0.090 (3)	0.114 (4)	0.244 (6)	−0.034 (3)	−0.006 (3)	−0.019 (4)
O7	0.054 (2)	0.119 (3)	0.088 (2)	0.0052 (19)	0.0086 (18)	−0.010 (2)
N3	0.056 (3)	0.097 (4)	0.066 (2)	−0.005 (2)	0.017 (2)	−0.007 (2)
O8	0.072 (2)	0.089 (2)	0.115 (3)	−0.0035 (18)	0.0242 (19)	−0.0212 (19)

### Geometric parameters (Å, °)

**Table:**

Ag1—N1	2.126 (3)	C10—H10	0.9300
Ag1—N2	2.128 (3)	C11—C12	1.368 (5)
O1—C3	1.366 (4)	C11—H11	0.9300
O1—H1A	0.8200	C12—H12	0.9300
O2—C6	1.377 (4)	C13—C14	1.374 (5)
O2—C7	1.411 (4)	C13—H13	0.9300
O3—C22	1.377 (4)	C14—C15	1.374 (5)
O3—H3	0.8201	C14—H14	0.9300
O4—C19	1.373 (4)	C15—C16	1.370 (5)
O4—C18	1.411 (5)	C15—C18	1.491 (5)
N1—C11	1.328 (5)	C16—C17	1.375 (5)
N1—C10	1.330 (5)	C16—H16	0.9300
N2—C17	1.321 (5)	C17—H17	0.9300
N2—C13	1.323 (5)	C18—H18A	0.9700
C1—C2	1.369 (5)	C18—H18B	0.9700
C1—C6	1.384 (5)	C19—C20	1.368 (5)
C1—H1	0.9300	C19—C24	1.379 (5)
C2—C3	1.380 (5)	C20—C21	1.382 (5)
C2—H2	0.9300	C20—H20	0.9300
C3—C4	1.371 (5)	C21—C22	1.362 (5)
C4—C5	1.376 (5)	C21—H21	0.9300
C4—H4	0.9300	C22—C23	1.377 (6)
C5—C6	1.373 (5)	C23—C24	1.376 (5)
C5—H5	0.9300	C23—H23	0.9300
C7—C8	1.499 (5)	C24—H24	0.9300
C7—H7A	0.9700	O5—N3	1.206 (5)
C7—H7B	0.9700	O6—N3	1.176 (5)
C8—C12	1.364 (5)	O7—N3	1.251 (5)
C8—C9	1.394 (5)	O8—H31	0.8500
C9—C10	1.367 (5)	O8—H32	0.8499
C9—H9	0.9300		
			
N1—Ag1—N2	167.10 (13)	C8—C12—C11	119.7 (4)
C3—O1—H1A	109.5	C8—C12—H12	120.2
C6—O2—C7	117.6 (3)	C11—C12—H12	120.2
C22—O3—H3	109.5	N2—C13—C14	124.3 (4)
C19—O4—C18	117.3 (3)	N2—C13—H13	117.8
C11—N1—C10	116.8 (3)	C14—C13—H13	117.8
C11—N1—Ag1	121.6 (3)	C15—C14—C13	119.0 (4)
C10—N1—Ag1	121.5 (3)	C15—C14—H14	120.5
C17—N2—C13	116.1 (4)	C13—C14—H14	120.5
C17—N2—Ag1	127.1 (3)	C16—C15—C14	117.1 (4)
C13—N2—Ag1	116.6 (3)	C16—C15—C18	120.7 (4)
C2—C1—C6	120.8 (4)	C14—C15—C18	122.1 (4)
C2—C1—H1	119.6	C15—C16—C17	119.9 (4)
C6—C1—H1	119.6	C15—C16—H16	120.1
C1—C2—C3	120.2 (4)	C17—C16—H16	120.1
C1—C2—H2	119.9	N2—C17—C16	123.5 (4)
C3—C2—H2	119.9	N2—C17—H17	118.2
O1—C3—C4	117.6 (3)	C16—C17—H17	118.2
O1—C3—C2	123.5 (4)	O4—C18—C15	109.1 (3)
C4—C3—C2	118.8 (4)	O4—C18—H18A	109.9
C3—C4—C5	121.3 (4)	C15—C18—H18A	109.9
C3—C4—H4	119.3	O4—C18—H18B	109.9
C5—C4—H4	119.3	C15—C18—H18B	109.9
C6—C5—C4	119.8 (4)	H18A—C18—H18B	108.3
C6—C5—H5	120.1	C20—C19—O4	125.0 (4)
C4—C5—H5	120.1	C20—C19—C24	119.1 (4)
C5—C6—O2	124.8 (3)	O4—C19—C24	115.8 (4)
C5—C6—C1	119.1 (4)	C19—C20—C21	120.3 (4)
O2—C6—C1	116.2 (3)	C19—C20—H20	119.9
O2—C7—C8	108.4 (3)	C21—C20—H20	119.9
O2—C7—H7A	110.0	C22—C21—C20	120.4 (4)
C8—C7—H7A	110.0	C22—C21—H21	119.8
O2—C7—H7B	110.0	C20—C21—H21	119.8
C8—C7—H7B	110.0	C21—C22—C23	120.0 (4)
H7A—C7—H7B	108.4	C21—C22—O3	122.7 (4)
C12—C8—C9	117.4 (4)	C23—C22—O3	117.3 (4)
C12—C8—C7	123.5 (4)	C24—C23—C22	119.5 (4)
C9—C8—C7	119.0 (3)	C24—C23—H23	120.2
C10—C9—C8	119.0 (4)	C22—C23—H23	120.2
C10—C9—H9	120.5	C23—C24—C19	120.7 (4)
C8—C9—H9	120.5	C23—C24—H24	119.6
N1—C10—C9	123.5 (4)	C19—C24—H24	119.6
N1—C10—H10	118.2	O6—N3—O5	120.7 (5)
C9—C10—H10	118.2	O6—N3—O7	118.3 (5)
N1—C11—C12	123.6 (4)	O5—N3—O7	121.0 (5)
N1—C11—H11	118.2	H31—O8—H32	109.0
C12—C11—H11	118.2		

### Hydrogen-bond geometry (Å, °)

**Table:**

*D*—H···*A*	*D*—H	H···*A*	*D*···*A*	*D*—H···*A*
O1—H1A···O8^i^	0.82	1.90	2.661 (4)	155
O3—H3···O7	0.82	1.88	2.698 (5)	176
O8—H31···O7	0.85	2.05	2.885 (5)	167
O8—H32···O3^ii^	0.85	2.00	2.833 (4)	165

Symmetry codes: (i) −*x*+2, *y*+1/2, −*z*+3/2; (ii) −*x*−1, *y*−1/2, −*z*+1/2.
